# Mothers’ Stress as a Predictor of Preschoolers’ Stress in the Context of Parental Practices

**DOI:** 10.3390/children11111274

**Published:** 2024-10-22

**Authors:** Natalia A. Rudnova, Dmitriy S. Kornienko

**Affiliations:** Federal Scientific Center of Psychological and Multidisciplinary Research, Moscow 125009, Russia; dscorney@mail.ru

**Keywords:** perceived stress of children, perceived stress of mothers, positive parenting, punitive parenting, inconsistent parenting

## Abstract

Background/Objectives: Over the past decade, there has been a significant increase in distress among children, as well as in the long-term effects of childhood stress. Parents, particularly mothers, are the most important social partners for preschool-aged children and may help protect them from stress. The current study aims to investigate how parental practices impact children’s perceived stress in relation to their mothers’ stress. Methods: The sample comprised 346 mothers of preschoolers aged 20 to 48 years (M = 34.39, SD = 6.16). Participants completed an online survey that included the Perceived Stress Scale for Parents of Preschoolers, the Perceived Stress Scale, the Alabama Parenting Questionnaire—Preschool Revision, and questions regarding their socio-demographic characteristics. Results: Punitive parenting contributed positively, while positive parenting contributed negatively to children’s distress. When mothers’ perceived stress was taken into account, positive parenting lost its significance. Punitive parenting and maternal perceived stress were the only positive predictors of child distress. Conclusions: Stress in mothers may lead to a decrease in their involvement in the upbringing and parenting of preschoolers, potentially contributing to increased stress levels in children. Intervention programs aimed at reducing preschoolers’ stress should incorporate classes for parents that also could be focused on stress regulation for parents and training in positive parenting practices.

## 1. Introduction

A significant increase in distress levels and mental health crises among children has been observed by researchers over the past decade [1]. Various social, economic, and political processes impact the lives of adults, youth [2], and children alike [3]. Many of these events induce children’s stress and affect their brain maturation and growth [4], as well as their cognitive, regulatory, and emotional development [5].

Despite numerous approaches, studies on stress primarily focused on the stress experienced by adults and adolescents [6], while the issue of stress in children has remained largely overlooked for a considerable period. However, the COVID-19 pandemic has highlighted a huge gap in our understanding of children’s stress and has prompted a rapid increase in research addressing this issue [7,8]. Earlier research has already accumulated evidence demonstrating the long-term consequences of childhood stress [9,10,11]. Contemporary knowledge regarding the children’s stress encompasses various definitions and classifications, including physical, psychological, and perceived stress; chronic and acute stress; traumatic and toxic stress [12]. Numerous methods for investigating children’s stress have been developed [13,14,15]. Additionally, a multitude of studies aim to identify significant predictors of children’s stress. Given the age-related features of preschoolers, one of the most critical factors influencing children’s stress is family and parental characteristics.

The social buffering approach assumes that the presence of a supportive figure can protect children from stress or reduce their stress levels [16,17]. Thus, this approach highlights the importance of parent or caregiver well-being and the features of relationships with children [18,19,20]. Therefore, the current study aims to examine the impact of various dimensions of parenting on children’s stress, with a particular focus on maternal stress.

### 1.1. Stress of Preschoolers

Previous studies have identified age-related features of stress in preschoolers. It is important to note that children’s ability to recognize, differentiate, and label psychological states is underdeveloped due to the limited maturation of their cognitive and emotional resources [21,22,23]. This finding has two significant implications for understanding children’s stress indicators and measuring stress based on these features.

First, under stress, children often exhibit their emotional state through a physical or behavioral manifestation, and various signs of children’s stress have been documented. Preschoolers may display regressive behavior such as thumb- or finger-sucking, lip-biting, and nail-gnawing. They might also report physical complaints, including stomachache or headache, and experience sleep disturbances such as difficulty falling asleep, apnea, bruxism, and waking up during the night [24]. Nevertheless, psychological or emotional signs may also be observed. Children under stress may show a decrease in concentration, a high rate of irritability, and an increase in annoyance, along with conflicts and mood swings. They may exhibit tendencies towards loneliness and isolation, or, in contrast, seek attention from their parents or caregivers and display a fear of separation [25,26].

Second, children’s self-reported feelings of stress are still developing, leading researchers to suggest that self-reporting may not be a suitable method for this group of respondents [27]. In this case, various alternative methods of stress investigation could be useful. Physiological stress may be assessed through objective measures such as heart rate variability, respiratory rate, blood pressure, body temperature, galvanic skin response, cortisol level, and others [13]. Another way of collecting data on children’s stress is by involving projective methods. For example, children could be asked to depict a human, a non-existent animal, or their family. Also, a test could involve having the child select appropriate responses to a picture or to complete a sentence [28]. Observation of children’s behavior by researchers could also present information about the rate of stress [29]. However, one of the most commonly used methods for assessing children’s stress is through reports from parents or caregivers. Previous studies showed that adults can provide reliable information about their children’s stress experience [30].

### 1.2. Role of Parental Characteristics for Stress of Children

The social buffering approach suggests that the presence of a significant person during a stressful situation may help an individual to cope [31,32]. The hypothalamic–pituitary–adrenocortical (HPA) system is activated in response to stress in the face of novelty, uncertainty, and other psychological stressors. However, prolonged activation of this system may lead to different negative outcomes and pathologies. Research has shown that the presence of a partner can reduce the individual’s cortisol response. While the mechanism underlying this effect is still under investigation, evidence suggests that the presence of a companion triggers activity in the prefrontal cortex and increases the level of oxytocin [33]. Specifically, an individual’s recognition of a companion’s presence and the related cortical processes moderate HPA responses to stressors. At the same time, social interactions, especially physical ones like touching and hugging, provoke oxytocin activity, which further diminishes HPA responses. Therefore, in the context of children’s stress investigation, it is important to continue exploring the role of parents, particularly mothers, as the most significant social partners of the child in preschool age.

The significance of parental involvement in children’s adaptation during stressful or traumatic events was established after World War II. Research indicates that children separated from their families demonstrated a higher level of trauma and long-term negative consequences compared to those who remained with their families, even in unsafe environments [34]. This data highlights the crucial role parents play in shaping children’s stress experience. Since that time, many different parental practices have been identified [35], and much evidence of the positive contribution of parents’ behavior and parent–child relationships on reducing child stress has been documented. For example, studies conducted within the framework of Bowlby’s attachment theory have shown that children with secure attachment and more sensitive caregivers tend to exhibit higher levels of optimism, self-reliance, and self-confidence, as well as improved attention, concentration, and emotional regulation. These characteristics are laid down in early childhood, and as inner resources help to cope with stress [36]. Conversely, parental withdrawal, neglect, abuse, and separation can lead to brain changes that have far-reaching outcomes for preschoolers’ social skills and stress regulation [34]. Contemporary studies of children’s stress in relation to parenting styles have also identified a positive association between the stress of children and punitive parenting, and a negative one with warm and supporting parenting [37]. Based on these results, we may suggest that positive parenting, which includes involving parents in upbringing, warmth, and emotional support of children, could be a predictor of low-level children’s stress, while negative parenting could raise it.

On one hand, parents must shield their children from unfavorable situations and provide them with tools to manage stress, but they may also experience stress themselves [38]. An investigation of post-traumatic stress disorders in children and their parents showed that parents may demonstrate various types of behavior as a result of their own stress. Along with positive scenarios, parents could overprotect their children, ignore negative experience or remain silent. They may also lose their functional abilities and control, reverse family roles (a child became a caretaker), or induce feeling of guilt and shame in their children by blaming them for what has occurred [34]. All these negative options have a detrimental effect on children’s development and aggravate their negative experience.

Data regarding the contribution of parents’ perceived stress on their children’s stress are limited. Some studies show that parental stress is associated not only with negative parenting practices but also with children’s behavioral problems [39], as well as internalizing and externalizing symptoms during childhood and adolescence [40,41]. Summarizing the existing information, we assume that parents in unstable and high-stress situations possess fewer resources for self-regulation and for providing support to their children [42]. Consequently, when parents experience stress, their children’s stress levels are likely to be higher.

### 1.3. Current Study

The current study aims to investigate how children’s perceived stress is influenced by positive, punitive, and inconsistent parenting in relation to their mothers’ stress.

Existing data about associations between the perceived stress of children and their mothers and parenting dimensions allow us to propose the following hypotheses: (1) the perceived stress of mothers relates to their parenting practices; (2) parenting makes a significant contribution to perceived stress of children: negative parenting (punitive and inconsistent) would increase the perceived stress level of children, while positive parenting would decrease it; (3) mother’s perceived stress serves as a positive predictor of the children’s stress.

## 2. Materials and Methods

### 2.1. Participants and Procedure

The sample comprised 346 mothers of preschoolers aged 20 to 48 years (M = 34.39, SD = 6.16). In total, 73% of the participants were married, 73% held a high level of education, and 51% were employed full-time. Among the mothers, 55% had only one child, 35% had two children, and the others had three or more children. The preschoolers were aged 4 to 7 years (M = 4.97, SD = 1.06); in total, 57% were male and 43% were female.

Participation in the study was voluntary; researchers received written consent from all respondents. Data collection was conducted through an online survey. The study materials were presented to the participants in the official language of the Russian Federation. All research procedures followed the ethical standards of the Russian Psychological Society.

### 2.2. Measures

The Perceived Stress Scale for Children (PSS-C) was developed for the investigation of child stress. The original version of the scale includes 13 items and is used as a single scale [14], while the Russian version has 10 items and demonstrates a two-factor structure with the Distress Scale and the Scale of Well-Being [15]. In the current study, the Distress Scale of the Russian version of PSS-C was adopted for the evaluation of the level of preschoolers’ stress by parents. It includes six items that assess different dimensions of stress that the child demonstrated last week (rushing or hurrying, worrying about being too busy, feeling angry, etc.). Participants rated every item on a Likert scale from 1 (“never”) to 4 (“very often”). Fit indexes of the Distress Scale: (χ2 (9) = 40.57, *p* < 0.001; CFI = 0.902; TLI = 0.837; SRMR = 0.05; RMSEA = 0.10, 95% CI [0.07:0.13]), Cronbach’s alpha is 0.69, CI [0.63:0.72].

The Perceived Stress Scale was utilized for the identification of the general level of stress among mothers. The current study used the Russian version of the Perceived Stress Scale, which has four items (PSS-4) [43] and is grounded in the original version of the Perceived Stress Scale [44]. Participants were asked to assess how often in the last month they felt that they were unable to control the important things in their lives and could not cope with all the things that they had to do, or that they felt confident about their ability to handle their personal problems and that things were going their way. A Likert scale from 1 (“never”) to 4 (“very often”) was used for participants’ answers. Fit indexes of the PSS-4: (χ2 (2) = 11.38, *p* < 0.01; CFI = 0.916; TLI = 0.749; SRMR = 0.04; RMSEA = 0.11, 95% CI [0.06:0.19]), Cronbach’s alpha is 0.57, CI [0.51:0.64].

The Alabama Parenting Questionnaire—Preschool Revision (APQ—PR) was used for the assessment of dimensions of parenting characteristics [45]. The Russian version of APQ-PR [46] includes 17 items that form three scales: Positive parenting, inconsistent parenting, and punitive parenting. Positive parenting is supporting and involving the parent in the child’s life (e.g., “you compliment your child when he/she does something well”); inconsistent parenting describes failure to observe discipline, promises, and rules by the parent (e.g., “you get so busy that you forget where your child is and what he/she is doing”); punitive parenting is the frequency of using different types of punishment as acceptable methods of parenting (e.g., “you slap your child when he/she has done something wrong”). Participants use a Likert scale from 1 (“never”) to 5 (“always”). Fit indexes of the APQ-PR: (χ2 (116) = 394.93, *p* < 0.001; CFI = 0.818; TLI = 0.787; SRMR = 0.09; RMSEA = 0.08, 95% CI [0.074:0.092]), Cronbach’s alpha for positive parenting is 0.79, CI [0.78:0.83], for inconsistent parenting is 0.55, CI [0.46:0.61], and for punitive parenting is 0.75, CI [0.71:0.79].

## 3. Results

Preliminary analysis of the descriptive statistics of study variables (Table 1) revealed that mothers evaluate their perceived stress and distress of children at a moderate level (M = 1.91 and M = 1.95, respectively). However, the range of responses and the standard deviation for the distress of children scale is narrower (minimum—1.17 and maximum—3.50, SD = 0.47) compared to the mothers’ perceived stress scale (minimum—1.00 and maximum—4.00, SD = 0.51). Among the parenting characteristics, positive parenting demonstrates the highest mean score (M = 4.53), while punitive parenting scored lower (M = 2.05) than inconsistent parenting (M = 2.93). At the same time, range of responses for positive parenting is narrower (minimum—2.29 and maximum—5.00) than that for punitive parenting (minimum—1.00 and maximum—4.33) or inconsistent parenting (minimum—1.00 and maximum—5.00).

The Shapiro–Wilk test found that the distributions of the investigated variables are not consistent with the normal distribution (*p* < 0.001).

Correlation analysis (Spearman’s rho) showed that distress of children has a positive association with perceived stress of mothers (r = 0.21, *p* < 0.001), punitive parenting (r = 0.21, *p* < 0.001), inconsistent parenting (r = 0.11, *p* < 0.05), and a negative one with positive parenting (r = −0.12, *p* < 0.05). Perceived stress of mothers has a negative association with positive parenting (r = −0.21, *p* < 0.001), a positive correlation with punitive parenting (r = 0.19, *p* < 0.001), and inconsistent parenting (r = 0.12, *p* < 0.05), while punitive and inconsistent parenting have a positive one (r = 0.31, *p* < 0.001). Punitive Parenting also has a negative correlation with positive parenting (r = −0.29, *p* < 0.001) (Table 2).

Multiply regression analysis was conducted to identify predictors of the distress of children based on maternal characteristics—perceived stress and parenting features. Distress of children was included in the regression models as a dependent variable; parenting characteristics were added on the first step, and as an independent variable, perceived stress of mothers was added on the second step. In the first step, the model explains 8% of the variance; in the second one, it explains 10% of the variance. All results of the regression analysis are presented in Table 3.

According to the results, the first model indicates that punitive parenting had a positive contribution (b = 0.14, *p* < 0.001), and positive parenting had a negative contribution (b = −0.11, *p* < 0.05) to the distress of children. In the second model, when the perceived stress of mothers was included, positive parenting lost its significant contribution and the only positive predictors of distress of children were punitive parenting (b = 0.13, *p* < 0.001) and perceived stress of mothers (b = 0.13, *p* < 0.01).

## 4. Discussion

Based on the results of the hypotheses testing, we may conclude that the perceived stress of mothers is negatively associated with positive parenting and positively linked with punitive and inconsistent parenting. The regression analysis showed that punitive parenting and maternal stress contribute to increased children’s stress, while positive parenting decreases it. Although inconsistent parenting did not significantly contribute to the children’s stress, a negative correlation was found. In general, these results accord with previous findings [47]. Additionally, we have an interesting finding regarding the diminished of a significance of positive parenting in the regression model where mother’s stress was added on the second step.

In general, the current study results reported that a mother’s stress negatively impacts parenting and children’s emotional state. This fact may be explained by the following—positive parenting requires certain inner resources from parents, such as time, patience, attention to their children, and a willingness to engage in interactions. When under stress, these inner resources are wasted on parents’ adaptation, self-regulation, and coping. That is why under stress, child–parent relationships could become less positive, leading parents to use disciplinary practices frequently. Considering that under stress not all parents able to cope their stress effectively [48], inconsistent parental behavior in such case is not uncommon, although it may not play a significant role. In turn, these changes in parental attitude and behavior may lead to the arising of children’s stress. Similar findings have been described in previous studies; for example, parental stress has been positive associated with children’s anxiety and behavior problems as well as with authoritarian parenting style [39] or physical disciplinary practices [49].

The current study also showed that mothers did not report extremely low and high levels of children’s stress. We could suggest that the lack of minimal values may imply that preschoolers are experiencing some stress, but the absence of maximal values suggests that this stress is not severe. These results are in line with current data on children’s well-being [50].

Despite the detrimental effect of maternal stress and punitive parenting on children’s stress, most respondents describe their attitude toward their children as positive, which aligns with previous findings [46]. Mothers who express confidence in their roles as engaged parents—emphasizing their strong relationships, frequent communication, and warm interactions with their children—believe that they provide adequate support for their children, rarely resort to different disciplinary practices, and strive for consistency in their rules [51]. Interestingly, the level of stress of both mothers and children appears to be nearly equal, and these variables demonstrate a positive correlation. We may speculate that positive and close relationships between mothers and children could lead to equalization of their stress levels. Although this assumption needs further exploration, it is reasonable to anticipate that if mothers effectively cope with their stress, the stress level of their children may also decrease. Moreover, positive experiences in parental stress management with stress could be a protective factor for children and foster a new internal resource for positive parenting and children’s stress regulation.

## 5. Conclusions and Limitations

Study findings showed that mothers’ stress, which may increase due to destructive and unstable life circumstances, could lead to a reduction in the mother’s involvement in the upbringing and parenting of preschoolers. In the context of a high-stress life situation, increased maternal stress and changes in mother–child relationships may manifest through withdrawal, low support, or emotional detachment. Consequently, children may also experience elevated stress levels. For this reason, intervention programs aimed at reducing preschoolers’ stress should incorporate classes for parents that not only provide information about the stressors affecting children and techniques for managing children’s stress but also focus on stress regulation for parents and training in positive parenting practices.

The current study has some limitations. First, parents tend to underestimate the emotional and behavioral problems of their children [52], which is why our results should be interpreted with caution. Addressing this tendency among parents may be possible using assessments that capture children’s perceptions of their stress. Second, values of Cronbach’s alphas are quite low. Although sample size and using short versions of scales, which can have lower levels of reliability indexes and values of confidence intervals, make it sufficient for using data, such results make us cautious in interpretation. Next, regression analysis demonstrated a quite low value of regression coefficients. We may speculate that it could mean the presence of any other and more significant factors that affect children’s distress. For example, it might be some external characteristics of the environment (safety, poverty, etc.). In this case, the perspective of the investigation could be studying environmental dimensions that may influence stress among children. Then, due to the cross-sectional design, we cannot identify some mechanisms and characteristics of mothers’ and preschoolers’ stress rates. In particular, it is not clear what the stressor was, how long the mother–child dyad experienced stress, what the relationships between mother and child were prior to the stress, and how the rate of stress fluctuated—mother’s stress was raised, or children’s stress was reduced during a stressful situation. Future studies should consider employing a longitudinal design with comprehensive measures of stress.

## Figures and Tables

**Table 1:** Descriptive statistics of investigated variables.

Variables	Mean	SD	Skewness	Kurtosis	Min.	Max.
Distress (children)	1.95	0.47	0.75	0.19	1.17	3.50
Perceived Stress (mothers)	1.91	0.51	0.57	0.75	1.00	4.00
Positive Parenting	4.53	0.47	−1.92	6.40	2.29	5.00
Punitive Parenting	2.05	0.66	0.69	0.32	1.00	4.33
Inconsistent Parenting	2.93	0.72	−0.23	0.04	1.00	5.00

Note. SD—Standard Deviation, Min.—Minimum, Max.—Maximum.

**Table 2:** Result of correlation analysis.

Variables	1	2	3	4
1. Distress (children)	—			
2. Perceived Stress (mothers)	0.21 ***	—		
3. Positive Parenting	−0.12 *	−0.21 ***	—	
4. Punitive Parenting	0.21 ***	0.19 ***	−0.29 ***	—
5. Inconsistent Parenting	0.11 *	0.12 *	−0.05	0.31 ***

Note. * *p* < 0.05, *** *p* < 0.001.

**Table 3:** Result of regression analysis.

Variables	b	Standard Error	Beta	t	*p*
Model 1 R2 = 0.08 Adjusted R2 = 0.07 F (3 342) = 9.831, *p* < 0.001					
(Intercept)	1.98	0.29		6.86	0.001
Positive Parenting	−0.11	0.06	−0.10	−1.93	0.05
Punitive Parenting	0.14	0.04	0.19	3.47	0.001
Inconsistent Parenting	0.05	0.04	0.08	1.42	0.16
Model 2 R2 = 0.10 delta R2 = 0.02 Adjusted R2 = 0.09 F (4 341) = 9.275, *p* < 0.001					
(Intercept)	1.63	0.31		5.17	0.001
Positive Parenting	−0.08	0.06	−0.08	−1.37	0.17
Punitive Parenting	0.13	0.04	0.19	3.25	0.001
Inconsistent Parenting	0.04	0.04	0.07	1.27	0.20
Perceived Stress (mothers)	0.13	0.05	0.14	2.66	0.01

## Data Availability

The full dataset is available upon request to the corresponding author.
